# Supplementary material for: Identification of photoperiod-induced specific miRNAs in the adrenal glands of Sunite sheep (Ovis aries)
Source: Front Vet Sci. 2022 Jul 22;9:888207. doi: 10.3389/fvets.2022.888207 (PMC9354845; doi:10.3389/fvets.2022.888207)
Supplement: Supplementary file 1 [file Data_Sheet_1.ZIP › Supplementary file/miRDeep2 file.zip!/miRDeep2 file/Know_Structure/SP42A1/oar-mir-3955.pdf]

miRBase precursor : oar-mir-3955
Total read count : 405
oar-miR-3955-5p read count : 366
oar-miR-3955-3p read count : 23
remaining reads : 16

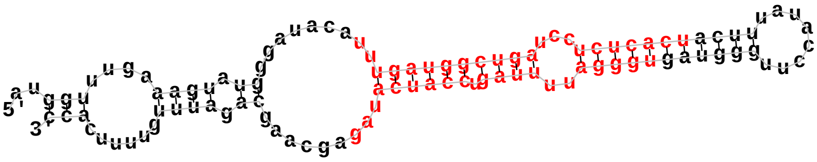

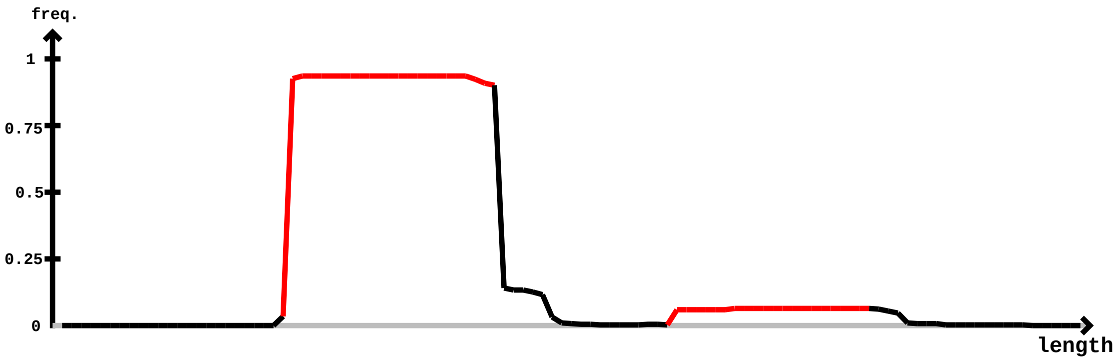

oar-miR-3955-5p

oar-miR-3955-3p

5'- augguuugaaaguaugggauacauuugauggcugauccucucacuacuuuauaccuuggguagugggauuuuaguccaucauagagcaagcagauuuguuuucacc -3'	exp		
...........((((...))))...((((((((((...(((((((((((........)))))))))))..)))).)))))).(((((((......))))))))....	reads	mm	sample
......................auuugauggcugauccucucacu...	12	0	seq
......................auuugauggcugauccucucacuacuuu..	2	0	seq
.......................uuugauggcugauccucuc..	5	0	seq
.......................uuugauggcugauccucuca...	6	0	seq
.......................uuugauggcugauccucucac..	3	0	seq
.......................uuugauggcugauccucucacu...	294	0	seq
.......................uuugauggcugauccucucacua..	3	0	seq
.......................uuugauggcugauccucucacuacu..	3	0	seq
.......................uuugauggcugauccucucacuacuu...	4	0	seq
.......................uuugauggcugauccucucacuacuuu..	32	0	seq
.......................uuugauggcugauccucucacuacuuua...	9	0	seq
.......................uuugauggcugauccucucacuacuuuau..	1	0	seq
.......................uuugauggcugauccucucacuacuuuauacc...	1	0	seq
........................uugauggcugauccucucacu...	2	0	seq
........................uugauggcugauccucucacuacuuuaua...	1	0	seq
........................uugauggcugauccucucacuacuuuauaccuugggua..	1	0	seq
....uagugggauuuuaguccaucauagagcaagc...............	1	0	seq
....ugggauuuuaguccaucauag......................	1	0	seq
....ugggauuuuaguccaucauaga.....................	3	0	seq
....ugggauuuuaguccaucauagag....................	3	0	seq
....ugggauuuuaguccaucauagagc...................	15	0	seq
....ugggauuuuaguccaucauagagca..................	1	0	seq
....uuuaguccaucauagagcaagc...............	1	0	seq
....uuuaguccaucauagagcaagcagauuuguu......	1	0	seq
